# An Investigation on the Total Thickness Variation Control and Optimization in the Wafer Backside Grinding Process

**DOI:** 10.3390/ma15124230

**Published:** 2022-06-15

**Authors:** Yuanhang Liu, Hongfei Tao, Dewen Zhao, Xinchun Lu

**Affiliations:** State Key Laboratory of Tribology, Tsinghua University, Beijing 100084, China; liuyuanh18@mails.tsinghua.edu.cn (Y.L.); thf20@mails.tsinghua.edu.cn (H.T.)

**Keywords:** total thickness variation, backside grinding, grinding tool configuration, angle adjustment, ultra-precision grinding

## Abstract

The wafer backside grinding process has been a crucial technology to realize multi-layer stacking and chip performance improvement in the three dimension integrated circuits (3D IC) manufacturing. The total thickness variation (TTV) control is the bottleneck in the advanced process. However, the quantitative analysis theory model and adjustment strategy for TTV control are not currently available. This paper developed a comprehensive simulation model based on the optimized grinding tool configuration, and several typical TTV shapes were obtained. The relationship between the TTV feature components and the spindle posture was established. The linear superposition effect of TTV feature components and a new formation mechanism of TTV shape were revealed. It illustrated that the couple variation between the two TTV feature components could not be eliminated completely. To achieve the desired wafer thickness uniformity through a concise spindle posture adjustment operation, an effective strategy for TTV control was proposed. The experiments on TTV optimization were carried out, through which the developed model and TTV control strategy were verified to play a significant role in wafer thickness uniformity improvement. This work revealed a new insight into the fine control method to the TTV optimization, and provided a guidance for high-end grinding tool and advanced thinning process development.

## 1. Introduction

In the three dimension integrated circuits (3D IC) manufacturing field, wafer stacking technology [1] has been developed as an extremely potential method to improve the integrity and computing performance of chips [2]. Out of the demand to realize more wafers stacking layer-by-layer, wafer needs to be thinned from about 780 μm to less than 10 μm and be processed to the status with a demanding thickness uniformity [3]. For a better chip thermo-mechanical reliability, the wafer needs to be processed to a proper thickness [4]. Except the high surface accuracy and integrity [5,6,7], the process efficiency and thickness uniformity are the main requirements for wafer thinning technology. In the industrial production, ultra-precision grinding technology with the workpiece self-rotation characteristic is commonly employed as a total thickness variation (TTV) controllable thinning process with high efficiency [8]. The TTV of the wafer must be controlled less than 1 μm in the advanced process [9]. It should be noted that the TTV control technology has been a crucial bottleneck for wafer backside grinding machine tool development and process improvement.

In the past decades, many researchers have focused on the theory and method of wafer backside grinding process. Tonshoff et al. [10] proposed an automatic adjustment of grinding tool spindle was necessary and the TTV of an 8 inch wafer was reduced to 1 μm. They just gave the experiment results, but the TTV control method and laws were not revealed. Sun et al. [11,12] defined the TTV shape components to make it more convenient for the TTV analysis. Tso et al. [13] focused on the kinematic model to predict wafer TTV and obtained the simulation results that the TTV could be influenced by the wheel infeed rate and the rotation speed ratio between the spindle and chuck table. They suggested that the inclination angle of the spindle needed to be determined appropriately to compensate the concave shape caused by the unsuitable process parameters. Sun et al. [14,15,16] systematically illustrated the process flow considering the chuck table dressing and wafer grinding, and introduced that the method to control TTV was similar to that for chuck table shape control. Their model predicted the variation trend of wafer TTV characteristic and chuck table shape, and qualitative experiments were conducted to validate the simulation results. Guo et al. [17,18] studied the numerical model for a novel grinding process with an outer rim remained on the wafer, to obtain the characteristics of grinding marks and TTV under the given process conditions. They pointed out that the inclination angles had great influence on the wafer TTV features. Tang et al. [19] revealed that the space angles had a significant effect on the wafer shape, while other process parameters could also have slight effect on it. However, it was hard to obtain a wafer flatness which was close to zero.

The effect of TTV control is closely related to the grinding tool configuration design [20]. Sun et al. [11] established the mathematic model to analyze how grinding tool configuration affected TTV shape components, and then proposed an optimized grinder configuration in terms of an easier operation for spindle angle adjustment, through which the “cross-talking” effect between the two TTV components could be eliminated. They pointed out an important criterion to reduce the difficulty of adjusting spindle inclination and achieving a desired TTV results, which required that only one of the TTV components could be affected when tilting the spindle axis around one of the adjustment axes. Zhu et al. [21] developed a two-spindle and three-workstation grinder based on the chuck topography model and the obliquity adjustment device. They adopted the grinder tool configuration which was similar to the optimized one proposed by Sun et al. [11], and they conducted the chuck dressing experiments to obtain a chuck shape as desired. However, more details about the wafer TTV control method and relevant technology requirements were not revealed in their study. Zhou et al. [22,23,24] developed a backside grinding machine with a horizontal mode. They also established a simulation model to obtain the wafer TTV profile transition laws at different tilt angle combinations. The experiments indicated that a significantly uniform thickness could be reached. Because they adopted the spindle configuration which might cause the “cross talking” effect between the two TTV components, the TTV adjustment mechanism was not revealed clearly.

Generally speaking, the above researchers mainly concentrate on the basic TTV numerical simulation model and obtain several typical TTV shapes under specific process conditions. To the best of our knowledge, no work has been reported to investigate the quantitative relationship between the TTV feature components and spindle inclination angles, so the control strategy for inclination automation adjustment has not been studied. At the same time, several typical grinder configurations and their influence on the TTV control have been compared and analyzed, but there are still more influence laws have not been revealed clearly, which are even different from the previous conclusions in the literature. Furthermore, although the influence factors of TTV are specific, the essential formation mechanism and change rules of TTV have not been revealed. For the requirements to industrial application, the key to realize TTV less than 1 μm is still unknown, including the strategy of TTV control and the requirements to the accurate inclination angle adjustment. These are also the essential points to the thinning process improvement and high-end equipment research and development.

## 2. The Mechanism of Angle Adjustment in the Wafer Backside Grinding Process

### 2.1. The Principle of Wafer Back Grinding Process

**Figure 1:**
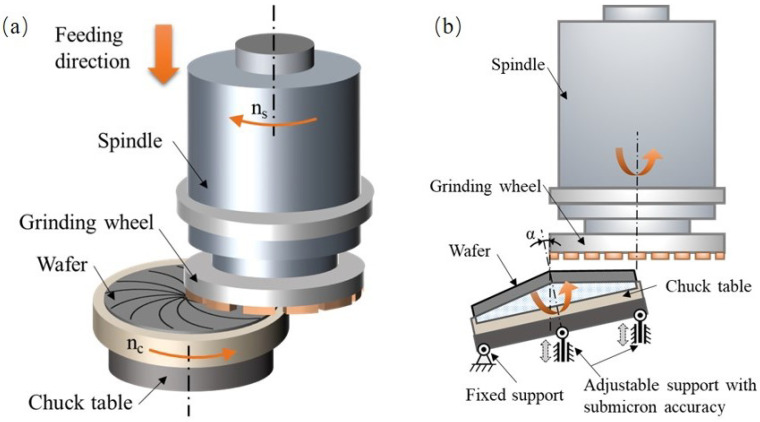
Illustration of wafer backside grinding. (**a**) Diagram of in-feeding mode grinding. (**b**) Principle of inclination angle adjustment for TTV control.

Figure 1 illustrates the principle of wafer backside grinding process. The wafer is mounted on a porous ceramics chuck table, and a cup-shaped grinding wheel is held on an aerostatic spindle. The spindle axis is offset by a distance relative to the chuck table axis to ensure that the wheel edge passes through the chuck table axis. The aerostatic bearings are used for both the spindle and chuck table to obtain high precision rotation accuracy and realize the material removal in the ductile mode [25,26,27]. In wafer backside grinding process, the spindle and chuck table rotate around their axes simultaneously, and the spindle feeds in the axial direction at a low speed, as shown in Figure 1a. The wafer backside material could be removed by the superimposed movements of the rotation and linear feeding.

Before processing the wafer, the chuck table needs to be ground to a cone shape, and the wafer is mounted on it, as shown in Figure 1b. The high accuracy screw mechanism is applied to adjust the inclination angles between the chuck axis and spindle axis, and thus compensate the TTV of the ground wafer. It should be noted that the control method of the chuck table shape is similar to the TTV control [28]. Therefore, this work mainly introduces the simulation model and experimental results on the TTV control. If necessary, the chuck table shape adjustment refers to the same theory and method proposed in this work.

### 2.2. The Effect of Grinding Tool Configuration on Angle Adjustment Method

**Figure 2:**
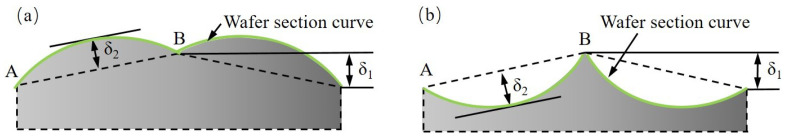
Typical TTV shape and its feature components. (**a**) Type 1: The superposition of cone shape and “M” shape. (**b**) Type 2: The superposition of cone shape and “W” shape.

As introduced in Section 2.1, the wafer rotates around the chuck table axis and the wheel rotates around the spindle axis. Therefore, a symmetrical wafer surface is formed by the enveloping motion of the grinding wheel edge. The TTV of wafer is usually resolved into two main feature components, i.e., $\delta_{1}$ and $\delta_{2}$ [11], as shown in Figure 2. In order to analyze the effect of the angle adjustment on the TTV, the values of $\delta_{1}$ and $\delta_{2}$ need to be quantitatively calculated. In Figure 2, $\delta_{1}$ is equal to the height difference between the wafer center and edge. Line segment AB is defined as a reference line, and $\delta_{2}$ could be obtained by calculating the maximal distance of all the points on wafer section curve to the reference line AB. The wafer TTV control is expected to realize by independently adjusting $\delta_{1}$ and $\delta_{2}$ without a coupling effect.

**Figure 3:**
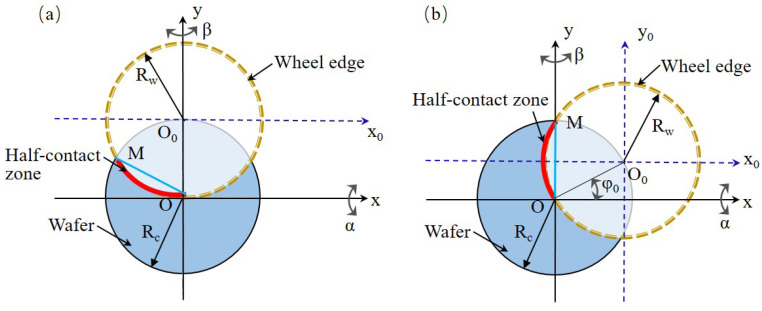
Diagram of classical grinding tool configurations. (**a**) Configuration 1: *x*-axis is not perpendicular to the arc OM. (**b**) Configuration 2: *x*-axis is perpendicular to the arc *OM*.

As mentioned above, the spindle axis is offset by a distance relative to the chuck table axis, but the layout between spindle and chuck table is not yet determined. The layout directly affects the angle adjustment strategy. The two TTV feature components are required to be adjusted individually, avoiding coupling change. Figure 3 displays two typical layout schemes of grinding machine, in which the red curve represents the wheel grinding edge. The configuration illustrated in Figure 3a was proposed by Tonshoff et al. [10]. The spindle posture adjustment is carried out by tilting angles around *x*-axis and *y*-axis. Because *x*-axis and *y*-axis are neither perpendicular nor parallel to the arc OM, $\delta_{1}$ and $\delta_{2}$ can not be adjusted and compensated individually, which has been verified by Sun et al. [11]. This means that it is pretty difficult to decrease TTV to a desirable state just through concise and effective operations. This work would not discuss it deeply.

In Figure 3b, angle adjustment direction is also set around *x*-axis and *y*-axis, but arc OM is perpendicular to *x*-axis and parallel to *y*-axis. Based on this layout, Sun et al. [11] established a simulation model and carried out some grinding experiments to study the wafer surface shape. They demonstrated that the angle adjustment along *x*-axis or *y*-axis only affects one of the wafer TTV features, and no coupling effect occurs between $\delta_{1}$ and $\delta_{2}$.

**Figure 4:**
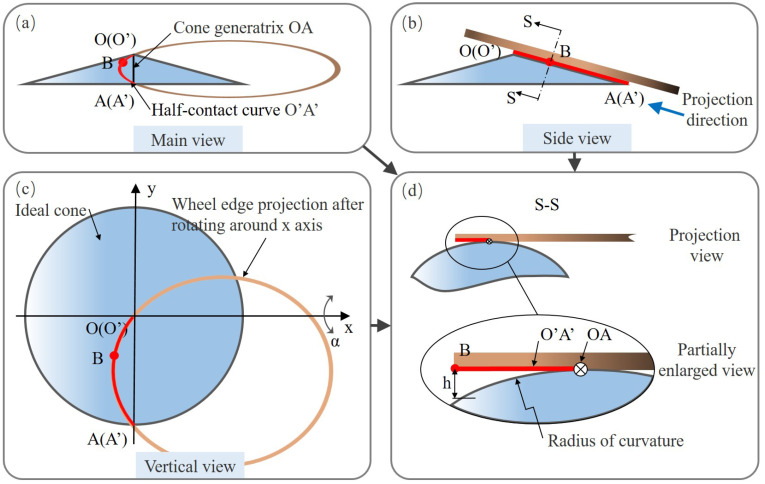
Geometrical schematic to analyze the envelope profile of wheel grinding edge after tilting around *x*-axis. (**a**) Main view. (**b**) Side view. (**c**) Vertical view. (**d**) Projection view.

Based on the nature of backside grinding process, the influence laws of space angles (α and β) on TTV feature components ($\delta_{1}$ and $\delta_{2}$) could be analyzed and understood more clearly. In fact, it is not completely consistent with the conclusions of the literature and more analysis detail refers to the subsequent contents. Figure 4 shows the three orthographic views of the spindle configuration shown in Figure 3b with the angle adjustment around *x*-axis. Point $O^{\prime }$ and point $A^{\prime }$ are the endpoints of the grinding edge curve. Coinciding with $O^{\prime }$ and $A^{\prime }$ individually, another two points, *O* and *A*, are set up to describe arc $O^{\prime }A^{\prime }$. An ideal cone is formed by the envelope of OA rotating around *z*-axis. Point *B* is an arbitrary point on the wheel grinding edge $O^{\prime }A^{\prime }$. The projection view is made from the direction shown in Figure 4b. As demonstrated in Figure 4d, there is a height difference from point *B* to the ideal cone surface. When point *B* coincides with point $O^{\prime }$ or $A^{\prime }$, the height difference tends to zero. This means that the grinding edge curve rotating around *z*-axis would form an approximate cone with a convex feature, different from the ideal cone. In other words, $\delta_{1}$ and $\delta_{2}$ would change simultaneously when only α changes, leading to a different conclusion compared with the existing literature [11]. Both chuck table dressing and wafer back grinding process are similar in the selected layout. Through a geometrical analysis for the grinding system, this work preliminarily discusses the new laws of how space angle α affects TTV features. Furthermore, the simulation model will be established to reveal practical TTV control and compensation laws.

## 3. The Wafer Shape Simulation Model and TTV Feature Analysis

### 3.1. The Mathematical Model for Ground Wafer Surface Shape

Based on the layout scheme illustrated in Figure 3b, a mathematical model for ground wafer surface shape is established to reveal the TTV control principle. The relationship between TTV feature components and space angles is obtained. It can be used for grinding tool design and processing parameter optimization to realize a good wafer thickness uniformity, such as TTV < 1 μm or lower.

As shown in Figure 3, the wafer circle with radius $R_{c}$ refers to the outer edge of the wafer. The grinding wheel circle with radius $R_{w}$ represents the outer edge of the grinding wheel. In order to describe the points on the wafer surface, two coordinate systems, i.e., O(XYZ) and $O_{0}$($X_{0}Y_{0}Z_{0}$), are established, and their origins are, respectively, set up at the centers of the wafer circle and grinding wheel circle. The wheel axis is almost parallel to the chuck table axis. Due to the requirement of TTV control in backside grinding process, the wheel spindle needs to incline around *x*-axis or *y*-axis to realize a semi-contact condition between the wheel grinding edge and wafer [29].

*OM* expresses the contact arc between wheel grinding edge and wafer, and its rotational movement around the *z*-axis in the O(XYZ) coordinate system generates the wafer surface shape. The coordinate of arc OM in the O(XYZ) coordinate system needs to be obtained when the posture of the spindle is adjusted to control the TTV features. The homogeneous matrix method effectively describes the coordinate transformation between O(XYZ) and $O_{0}$($X_{0}Y_{0}Z_{0}$) [30]. The position coordinate of the point on arc OM is denoted as ($x_{0}$, $y_{0}$, $z_{0}$) in $O_{0}$($X_{0}Y_{0}Z_{0}$). Based on the configuration in Figure 3, its corresponding point (x,y,z) in O(XYZ) is on the wafer surface and can be expressed as:

(1) $$\left[\begin{array}{c} x\\ y\\ z\\ 1 \end{array}\right]=T_{\alpha }\cdot T_{\beta }\cdot T_{xy}\left[\begin{array}{c} x_{0}\\ y_{0}\\ z_{0}\\ 1 \end{array}\right]$$

where matrices $T_{\alpha }$ and $T_{\beta }$, respectively, depict the tilt motions of arc OM around *x*-axis and *y*-axis; matrix $T_{xy}$ is used to deduce the coordinate translation transformation of arc *OM* along *x*-axis and *y*-axis. $T_{\alpha }$, $T_{\beta }$, and $T_{xy}$ are the common matrices in the homogeneous matrix method. All mentioned matrices can be given as:

(2) $$T_{\alpha }=\left[\begin{array}{cccc} 1 & 0 & 0 & 0\\ 0 & \cos\alpha & -\sin\alpha & 0\\ 0 & \sin\alpha & \cos\alpha & 0\\ 0 & 0 & 0 & 1 \end{array}\right]$$

(3) $$T_{\beta }=\left[\begin{array}{cccc} \cos\beta & 0 & \sin\beta & 0\\ 0 & 1 & 0 & 0\\ -\sin\beta & 0 & \cos\beta & 0\\ 0 & 0 & 0 & 1 \end{array}\right]$$

(4) $$T_{xy}=\left[\begin{array}{cccc} 1 & 0 & 0 & X_{0}\\ 0 & 1 & 0 & Y_{0}\\ 0 & 0 & 1 & Z_{0}\\ 0 & 0 & 0 & 1 \end{array}\right]$$

In Figure 3b, $\varphi_{0}$ is defined as the relevant angle between wheel circle and wafer edge circle, and can be derived as by

(5) $$\varphi_{0}=\arcsin\frac{R_{c}}{2R_{w}}$$

Then the position coordinate of ($X_{0}$, $Y_{0}$, $Z_{0}$) in O(XYZ) can be obtained by

(6) $$\left\{\begin{array}{cc} X_{0} & =R_{w}\cos\varphi_{0}\\ Y_{0} & =\frac{1}{2}R_{w}\\ Z_{0} & =0 \end{array}\right.$$

Meanwhile, arc OM in the $O_{0}\left(X_{0}Y_{0}Z_{0}\right)$ can be expressed as Equation (7). Through discretizing arc OM to lots of points and calculating their position coordinates in the O(XYZ), the wafer surface shape and TTV will be obtained accurately by

(7) $$\left\{\begin{array}{cc} x_{0} & =R_{w}\cos\left(\pi -\varphi_{0}+\varphi \right)\\ y_{0} & =R_{w}\sin\left(\pi -\varphi_{0}+\varphi \right)\\ z_{0} & =0 \end{array}\right.$$

where $0<\varphi <\arcsin\frac{R_{c}}{2R_{w}}$.

**Figure 5:**
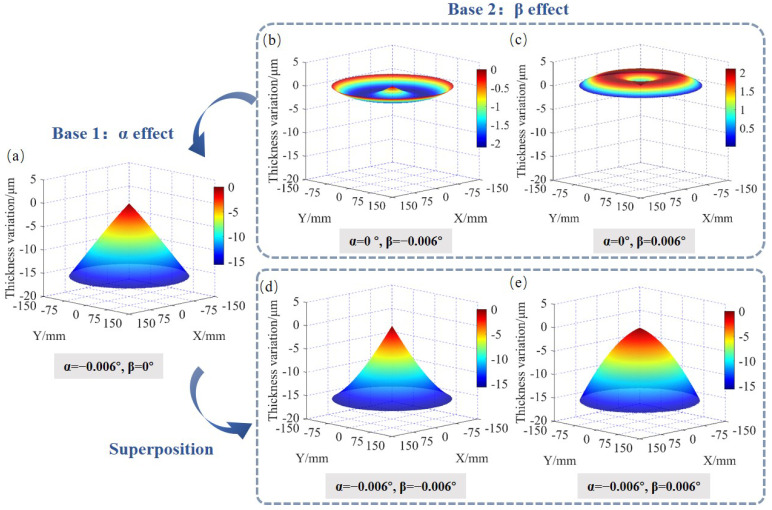
Wafer TTV simulation results and their topographies of under the typical space angle conditions. (**a**) α = −0.006° and β = 0°; (**b**) α = 0° and β = −0.006°; (**c**) α = 0° and β = 0.006°; (**d**) α = −0.006° and β = −0.006°; (**e**) α = −0.006° and β = 0.006°.

With the TTV simulation model described in the previous section, a program was developed using software MATLAB (The MathWorks, Inc., Natick, MA, USA). Figure 5 shows five typical wafer surface shapes obtained by the developed simulation model. According to the wafer TTV shape control model, the generatrix of wafer surface shape is almost straight when β = 0, which is also proved in Figure 5a. When α = 0, the conical degree will reduce to zero. Meanwhile, the shape presents a bulge or concave trend at *r* = 0.5$R_{w}$ when β>0 or β<0, which seems to be a ‘W’ or ‘M’ shape in Figure 5b,c. When neither α nor β is equal to zero, the wafer TTV shape presents a superposition effect due to the combined action of α and β, as shown in Figure 5d,e.

### 3.2. Effect of Angles Adjustment on TTV Features

**Figure 6:**
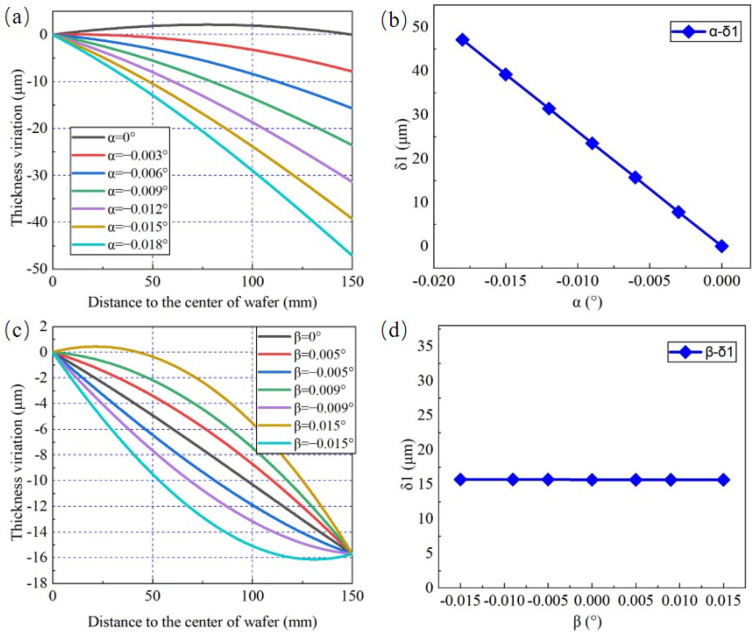
The change rules of $\delta_{1}$ affected by space angles. Effect of (**a**) α on the thickness variation (β = 0.006°); (**b**) α on $\delta_{1}$; (**c**) β on the thickness variation (α = −0.006°); (**d**) β on $\delta_{1}$.

First, the influence of adjustment angles α and β on the TTV feature component $\delta_{1}$ is analyzed. Figure 6a shows the thickness variation of the wafer under different values of α and the wafer TTV can be calculated by it. These curves describe the generatrix shapes of the wafers obtained in the preceding TTV simulation results. The TTV value is the difference between the maximum and minimum thickness, as shown on the curve. β is set to 0.006° as well as α ranges from −0.018° to 0°. With the increasing tilting angle of the wheel axis around *x*-axis, $\delta_{1}$ shows an upward trend, increasing from 0 to 46.9 μm, as displayed in Figure 6b. The thickness variation of wafer under different values of β is presented in Figure 6c. When β ranges from −0.015° to 0.015°, only the convex or concave degree relative to reference line of wafer shape varies, but the wafer $\delta_{1}$ keeps constant, equal to 15.7 μm, as shown as Figure 6d. The results show that $\delta_{1}$ merely depends on the adjustment angle of wheel axis around *x*-axis. In other words, $\delta_{1}$ could be controlled precisely only by adjusting α.

**Figure 7:**
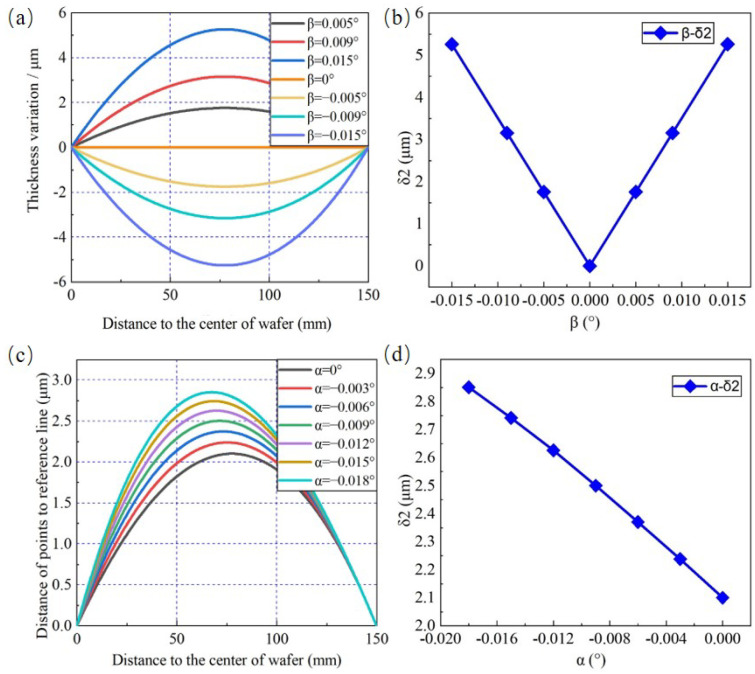
The change rules of $\delta_{2}$ affected by space angles. Effect of (**a**) β on the thickness variation (α = 0); (**b**) β on $\delta_{2}$; (**c**) α on the distance of points to the reference line (β = −0.006°); (**d**) α on $\delta_{2}$.

The relationship between wafer TTV feature component $\delta_{2}$ and β is analyzed below. Adjustment angle α is set to 0°, and β is set to 0°, ±0.005°, ±0.009°, and ±0.015°. As shown in Figure 7a, the wafer thickness variation shape is axisymmetric and presents an opposite convexity when β has the same value but the sign is reversed. Meanwhile, $\delta_{2}$ increases linearly with larger absolute value of β, as shown in Figure 7b. Figure 7c exhibits the wafer thickness variation shape under β is 0.006° and α varies from −0.018° to 0°, and $\delta_{2}$ can be further computed. With the increase in α, $\delta_{2}$ presents a decreased tendency shown in Figure 7d.

**Figure 8:**
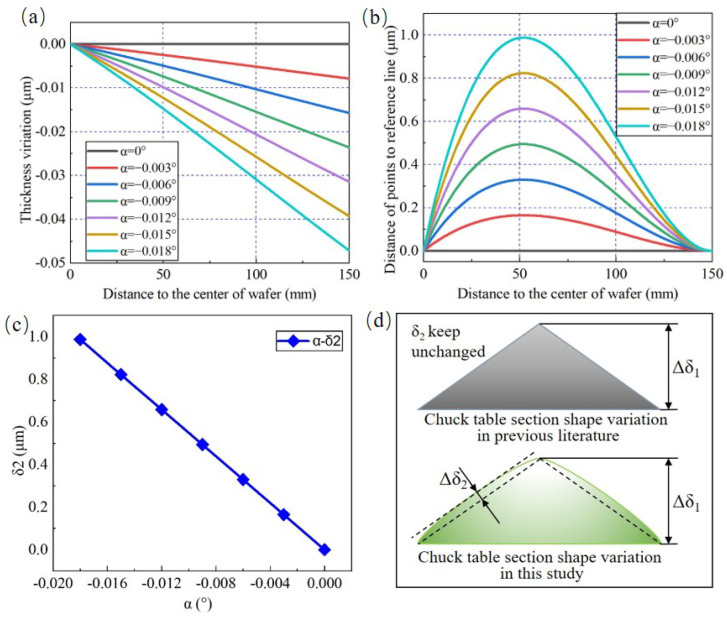
The change rules of $\delta_{2}$ affected by space angles. Effect of (**a**) α on the thickness variation (β = 0°); (**b**) α on the distance of points to the reference line (β = 0°); (**c**) α on $\delta_{2}$. (**d**) The qualitative analysis and comparison: the change rules of TTV features when α varies between the previous literature and this study.

In order to eliminate the influence caused by β on $\delta_{2}$ and only analysis the effect of α, β is set to 0° and α ranges from −0.018° to 0°. As shown in Figure 8a,b, the values of $\delta_{1}$ linearly tend to 46.9 μm, but the values of $\delta_{2}$ caused by α are lower than 1 μm and are much smaller than the values of $\delta_{1}$. As introduced in Section 2.2, $\delta_{2}$ can be calculated from the distance of all the points on the wafer section curve to the reference line shown in Figure 8b and it increases linearly with the increasing absolute value of α, illustrated in Figure 8c. However, the numerical simulation results shown in Figure 8b,c are different from the conclusion reported by [11,31]. They pointed out that $\delta_{2}$ would not be affected by α and the adjustment operation for α and β were absolutely independent, without a coupling effect. In Figure 8d, the comparison between the previous literature and this work is made. Actually, the wafer TTV feature component $\delta_{2}$ will be affected by both α and β simultaneously. Through both the accurate numerical simulation results in this section and figurative geometric qualitative analysis in Section 2.2, the relationship between wafer TTV features and adjustment angles could be understood more deeply.

### 3.3. Superposition Characteristics of TTV Feature Component

Because $\delta_{2}$ is affected by both α and β simultaneously, the superposition characteristics of α and β and quantitative relationship between them and $\delta_{2}$ need to be studied. It will be useful to analyze the wafer surface shape and make an effective strategy to control wafer TTV features. The wafer TTV is simulated under three different adjustment angle conditions as follows: 

Condition 1: α = 0°, β = 0.006°;

Condition 2: α = −0.006°, β = 0;

Condition 3: α = −0.006°, β = 0.006°.

**Figure 9:**
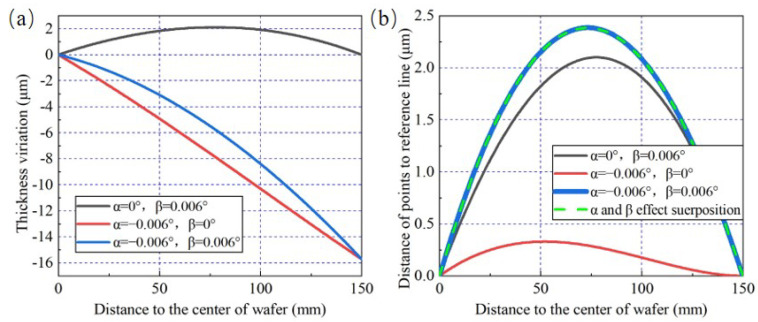
The verification to the superposition characteristics of $\delta_{2}$, under the combination of α and β (when β>0). (**a**) Thickness variation simulation results. (**b**) The calculation results of the distance of points to the reference line.

It can be observed from Figure 9a that the wafer thickness variation shape in condition 3 can be considered as the superposed one generated in conditions 1 and 2. Furthermore, $\delta_{1}$ is only affected by α, which could be verified directly here when comparing the wafer TTV under conditions 2 and 3. Under three conditions above, the different distances from points on wafer section curve to the reference line are calculated and shown in Figure 9b. The green dotted curve represents the linear superposition of distance curves under conditions 1 and 2. It is obvious that the green dotted curve is coincident with the blue curve. This result reveals that $\delta_{2}$ is finally generated by the effects of both α and β, and there is a clear linear superposition relationship among them.

Similarly, another groups of α and β combination condition are set to verify the superposition characteristic of $\delta_{2}$. The angle parameters are presented as follows: 

Condition 4: α = 0°, β = −0.006°;

Condition 5: α = −0.006°, β = 0;

Condition 6: α = −0.006°, β = −0.006°. 

**Figure 10:**
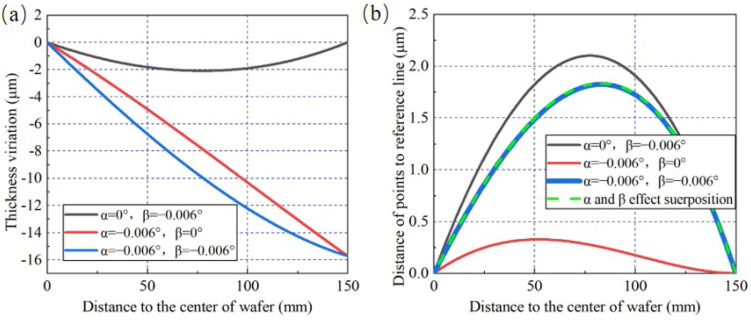
The verification to the superposition characteristics of $\delta_{2}$, under the combination of α and β (when β<0). (**a**) Thickness variation simulation results. (**b**) The calculation results of the distance of points to the reference line.

From Figure 10, the same conclusions can be conducted that $\delta_{2}$ is affected by both α and β. In Figure 10b, the blue curve presenting the condition 6 is coincident with the green dotted curve, which is obtained by the distance of points to the reference line under the condition 5 minus that of the condition 4. So $\delta_{2}$ under condition 6 could be calculated by a linear superposition of values caused by α and β separately. The diversity caused by the two groups of adjustment angle conditions is the $\delta_{2}$ value and the shape of distance superposition curve. When β = 0.006°, the distance curves generated by α and β are convex and the final $\delta_{2}$ value can be calculated by the sum of $\delta_{2}$ obtained by conditions 4 and 5. When β = −0.006°, the distance curve caused by α or β presents a contrary shape, which means that β leads to a concave curve compared to the convex curve generated by α. Therefore, the final $\delta_{2}$ value needs to be calculated by the difference between two $\delta_{2}$ components. The analysis results for two groups of typical adjustment angle conditions demonstrate that $\delta_{2}$ is affected by both β and α simultaneously, and can reflect their superposition characteristics.

### 3.4. TTV Control Strategy of Wafer Back Grinding

In this work, a simulation model has been established to analyze the quantitative relationship between the wafer TTV and adjustment angles. The wafer TTV depends on the two space angles (α and β) around the *x*-axis and *y*-axis between the wheel spindle and chuck table. The wafer TTV shape feature components, $\delta_{1}$ and $\delta_{2}$, are defined to conduct quantitative analysis. Through simulating several samples under some typical α and β combination conditions, wafer TTV features are calculated and analyzed. The wafer TTV features are the linear superposition effects caused by α and β, and can be controlled accurately by adjusting the two space angles.

According to the simulation and analysis results in Section 3.2, $\delta_{1}$ is only affected by α, so it is relatively concise to control $\delta_{1}$ by adjusting α. However, $\delta_{2}$ is affected by both α and β, an effective strategy needs to be proposed to obtain a desired wafer thickness uniformity. The universal principle for wafer TTV optimization is that:

1. If $\delta_{1}$ is greater than the desired value, the first step is to adjust α and make $\delta_{1}$ decrease, regardless of whether $\delta_{2}$ needs to be optimized or not. The second step is to adjust β to compensate for $\delta_{2}$ caused by both the $\delta_{1}$ adjustment and the original error of $\delta_{2}$.
2. If only $\delta_{2}$ needs to be reduced, just adjust β to compensate for $\delta_{2}$ to a desired value. Meanwhile, α must remain constant.

### 3.5. The Analysis of Actual Thinning Process and Related Technology Requirements

**Figure 11:**
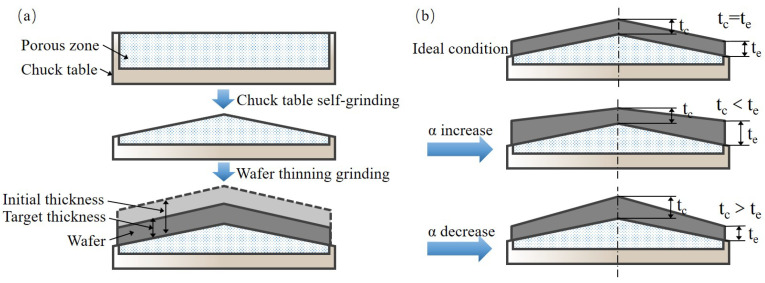
Diagram of the wafer thinning process flow. (**a**) Chuck table dressing and wafer grinding. (**b**) The wafer TTV variation trend caused by space angle variation (taking α and its influence for example).

In the actual grinding process, the upper surface of chuck table presents a cone shape after dressing. The wafer is adsorbed by a porous vacuum zone and thinned to a preset thickness, as shown in Figure 11a. As the method to control the chuck table shape by adjusting α and β is similar to that of wafer TTV, their adjusting accuracy demand is also the same.

The first step in the chuck table dressing process is to make the wheel spindle axis and chuck table axis parallel. Then we need to adjust α to control the cone height from the center to edge. The chuck table cone height needs to be measured and even re-dressed to the desired value during this process. Re-dressing work is based on the α adjustment, but the angle variation is relative to the current location rather than the parallel position mentioned above. The upper surface of chuck table will present a convex shape here. Finally, we should keep α stable and adjust β according to the measuring results of chuck table shape. In this way, $\delta_{1}$ is controlled first via changing α and remaining β stable. Then $\delta_{2}$ is controlled through adjusting β and keeping α stable, which can diminish the $\delta_{2}$ variation caused by the first step. Otherwise, the coupling variation of $\delta_{2}$ will happen.

The wafer thickness variation should be very close to zero theoretically after chuck table dressing because the space angles between the spindle and chuck table are not changed deliberately. However, the wafer thickness variation would deteriorate due to the various process parameters, as shown in Figure 11b, including grinding force [32], structure stiffness, grinding wheel type, and other factors. In the industrial production, TTV compensation is necessary and is always carried out according to the measurement results of $\delta_{1}$ and $\delta_{2}$ [33,34]. The adjustment laws of TTV features are the same as shown above. For example, $\delta_{1}$ needs to be compensated by adjusting α as well as $\delta_{2}$ caused by α variation needs to be concerned. The compensation of $\delta_{2}$ only needs to adjust β.

In the 3D IC industry, TTV lower than 1 μm is the technical tendency, and thus the adjustment accuracy of TTV feature components needs to be far lower than 1 μm. To meet the demand of TTV fine control, the adjustment accuracy of TTV feature components needs to be lower than 0.25 μm, and the adjustment accuracy of α and β needs to be lower than 0.0001°. The design and development of advanced grinding machines have to follow this technical specification or criterion.

## 4. Experimental Verification

### 4.1. Experimental Setup

**Figure 12:**
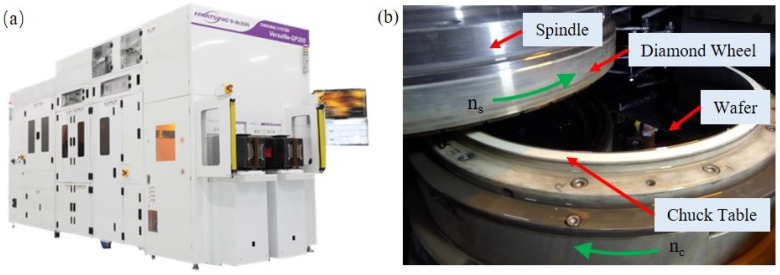
Diagram of the experiment setup. (**a**) The ultra-precision grinding machine tool: Versatile-GP300 [35]. (**b**) The internal structure when a wafer is mounted on the chuck table.

In order to verify the correctness of the developed model on chuck table shape and wafer TTV control, some grinding experiments, including chuck table dressing and wafer backside grinding, were carried out on the ultra-precision grinding machine tool (Hwatsing Technology Co., Ltd., Tianjin, China Versatile-GP300, developed by the authors’ team), as shown in Figure 12a. It should be emphasized that the optimized spindle and chuck table configuration has been adopted to Versatile-GP300. The chuck table contains a porous ceramics structure so that the 12 inch wafer can be mounted tightly on the upper surface of chuck table, as shown in Figure 12b.

**Figure 13:**
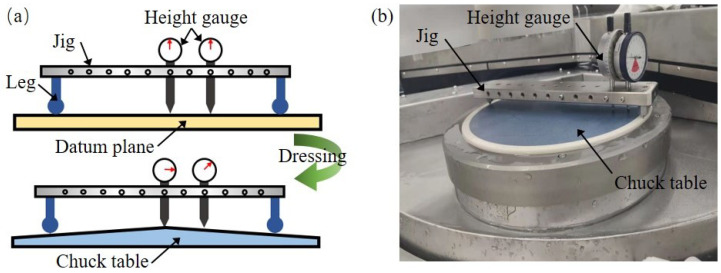
The device for chuck table shape measurement. (**a**) Diagram of the in situ measurement. (**b**) The actual measurement on the grinding tool.

The chuck table section shape could be measured in situ by the special measurement device shown in Figure 13a. This measurement device consists of a jig with three fixed legs and height gauges. The height gauge usually adopts a dial indicator or other displacement sensor. On the jig, several mounting holes for the dial indicator are made. The height differences between a specific point on the upper surface of chuck table and the datum plane can be measured. First, the jig with the height gauge is placed on a datum plane (a granite plate with the flatness lower than 1 μm), and the reading of the height gauge is recorded as a zero position. Then the height gauge is fixed on the jig, and the jig needs to be manually moved to the upper surface of the chuck table [36]. Because there is a height difference between the center and the edge of the chuck table, the reading of the height gauge will change and it needs to be recorded. The distances of these mounting holes center to jig center are 0 mm, 25 mm, 50 mm, 75 mm, 100 mm, 125 mm, and 150 mm, respectively, so the chuck table section shape could be obtained through multipoint measurement, as shown in Figure 13b.

**Figure 14:**
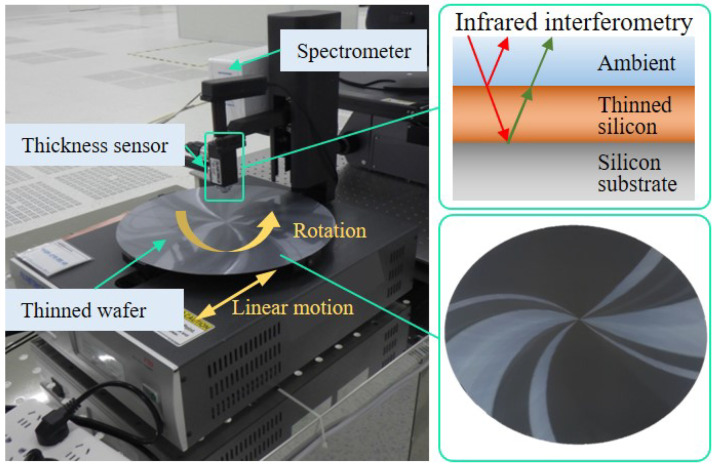
The device for wafer TTV measurement and its principle.

The wafer TTV is measured by the thin-film analyzer (F50, Filmetrics Inc., San Diego, CA, USA) as displayed in Figure 14. This instrument employs the principle of infrared interference to ensure a higher accuracy [37,38]. The measuring range and accuracy for silicon material are 7–1000 μm and 0.4% full scale, respectively. Chuck table dressing and wafer backside grinding experiments are carried out under the process parameters listed in Table 1. In the field of grinding technology, the symbol (#) with a number following (like #600, as shown in Table 1) is used to quantify the size of the abrasive grain in the grinding wheel. The size of the abrasive grain has been stated in Table 1.

### 4.2. Chuck Table Dressing

**Figure 15:**
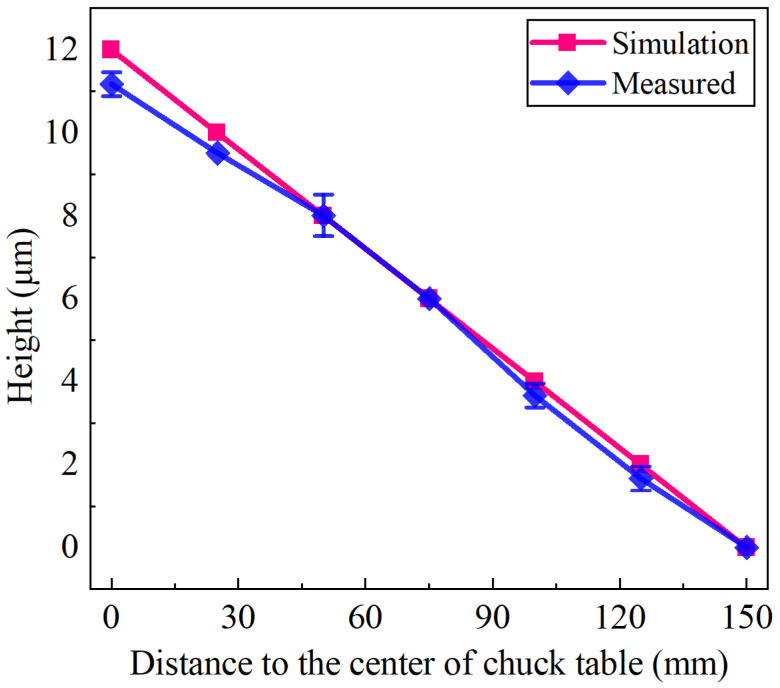
The comparison between the experimental and simulation results of the chuck table shape after dressing process.

In industrial production and application, the height difference from the chuck table center to the edge is always 10–15 μm, and a cone shape is desirable. So this work set α = 0.0048° (corresponding to 12 μm). Figure 15 shows the comparison between the simulated and measured results of chuck table shape. Although the experiment result presents a slight deviation from simulation results, it is still acceptable because their overall trend is consistent and the error is less than 1 μm. Meanwhile, the standard deviation for each data point is less than 1 μm. The experiment results match well with the simulation.

### 4.3. Wafer Back Grinding and TTV Optimization Control

**Figure 16:**
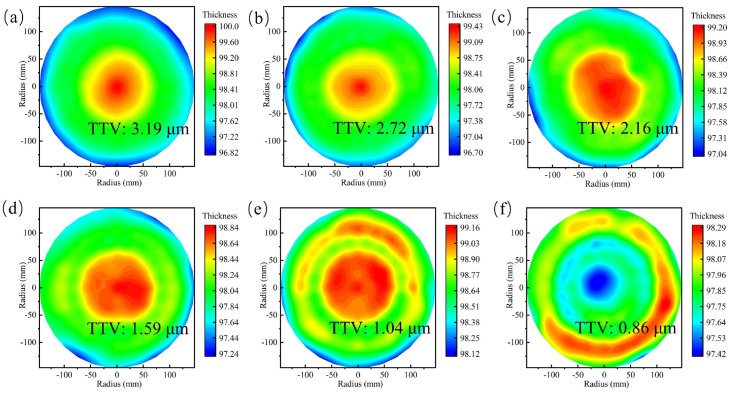
The TTV measurement results. (**a**) The original TTV just after the chuck table dressing; (**b**–**f**) TTV compensation results by adjusting α angle.

**Figure 17:**
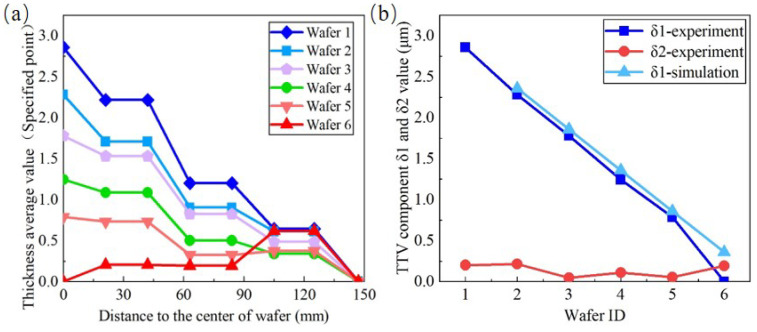
The quantitative analysis of TTV measurement results. (**a**) The thickness average values of points at the same radius. (**b**) The trends of $\delta_{1}$ and $\delta_{2}$ with the variation of α angle, and the comparison of $\delta_{1}$ between the experimental and simulation results.

The wafer backside grinding experiments were conducted after chuck table dressing. The wafer was thinned from 775 μm to about 100 μm under the processing conditions mentioned in Table 1. The thickness contours are displayed in Figure 16. Figure 16a shows the first piece of wafer thinned directly after chuck table dressing. Its TTV is 3.19 μm and different from the ideal condition. The reason has been explained in Section 3.5. To calculate $\delta_{1}$ and $\delta_{2}$, the measured data at the same radius (radius = 0 mm, 21 mm, 42 mm, 63 mm, 84 mm, 105 mm, 125 mm, and 147 mm) are averaged. For the first wafer, the values of $\delta_{1}$ and $\delta_{2}$ are 2.85 μm and 0.21 μm, respectively. The TTV of the first wafer presents a typical cone shape. The $\delta_{2}$ value is close to zero and does not need to be compensated at this step. Meanwhile, the thickness uniformity along the circumferential direction meets the technical requirement. Then another five pieces of wafer were thinned under different α angle variations set to −0.0002°, −0.0004, −0.0006°, −0.0008°, and −0.001° (negative angle clarifies the adjustment direction). The TTV contours are shown in Figure 16b–f. The thickness average is calculated similar to the first wafer, and their TTV profiles are exhibited in Figure 17a. Obviously, TTV values show a downward tendency, and the desired TTV, less than 1 μm, is obtained.

The values of $\delta_{1}$ and $\delta_{2}$ for each wafer are calculated and shown in Figure 17b. From the second to the fifth wafer, $\delta_{1}$ decreases linearly and matches well with the simulation results. Meanwhile, the value of $\delta_{2}$ for each wafer keeps fluctuating in a small range, because the both the original $\delta_{2}$ and $\delta_{2}$ variation caused by α are too slight. Many other factors, involving grinding heat, contact deformation, material plastic removal and so on, are not considered in this work. Thus, there exists a deviation of $\delta_{1}$ between the experimental and simulated data.

The values of α and β are controlled by the micro-motion mechanism, and its motion error would lead to an angle error directly affecting the TTV compensation accuracy. When TTV is less than 1 μm, the related process factors, such as chuck table surface cleaning, grinding heat, coolant, wafer surface initial planarization, machine stiffness, and so on, may be magnified and lead to adverse effects on this scale.

## 5. Conclusions

This paper focuses on the TTV control theory and method in the wafer backside grinding process. The numerical model is established to obtain wafer TTV shape under an optimized configuration scheme of grinding machine. Based on the simulation model, wafer TTV features can be quantified, and the effects caused by adjustment angles is further analyzed. Chuck table dressing and wafer backside grinding experiments are carried out to verify the accuracy of proposed models.

The wafer TTV shape is determined by the envelope movement of wheel grinding edge and affected by the superposition effect of adjustment angles (α and β). Two feature components ($\delta_{1}$ and $\delta_{2}$) are used to quantify the influence of α and β on wafer TTV. The results show that $\delta_{1}$ is only determined by α. Unlike the previous research, this paper demonstrates that both α and β can impact $\delta_{2}$ with the linear superposition. This conclusion reveals a new formation mechanism of TTV shape and provides a new insight into the fine control to the TTV optimization. Therefore, a more effective and accurate TTV control strategy can be given: (1) When only $\delta_{2}$ exceeds the desired value, only β needs to be adjusted. (2) When $\delta_{1}$ exceeds the allowable value, the principle is to preferentially reduce $\delta_{1}$ through adjusting α. Then $\delta_{2}$ needs to be checked and compensated by adjusting β, with the consideration including the original $\delta_{2}$ error and negative effect caused by α adjusting. The above steps may need to be repeated until neither $\delta_{1}$ nor $\delta_{2}$ exceeds the target value.

In the experiment for the chuck table dressing process, a cone-shape section is obtained as expected. The height difference from the center to the edge is 11 μm and matches well with the simulation analysis. The process results present that the shape of the chuck table can be controlled accurately by adjusting the posture of the spindle. Wafer backside grinding experiments reveal that the wafer TTV can be controlled accurately via adjusting α and β. The $\delta_{1}$ value is proved to decrease linearly with various α as expected. A wafer with TTV value of 0.86 μm is achieved, showing a good agreement with simulation results. In summary, this work not only provides meaningful guidance for machine structure design and configuration optimization in the wafer backside grinding process, but also gives an effective solution for improving wafer thickness uniformity to meet the more and more strict requirements in the 3D IC and advanced package field.

For the further research, TTV is required to be diminished to less than 0.5 μm or lower, so an auto-compensation system for TTV needs to be developed. This auto-compensation system may consist of the in situ TTV measurement device, intelligent recognition algorithms for the TTV features, and auto-adjustment device for controlling the posture of the spindle. With this auto-compensation system, the uniformity of the wafer, and the reliability of the grinding machine tool could be improved further. 

## Figures and Tables

**Table 1:** Process parameters in the experiments of chuck table dressing and wafer grinding.

Process Parameters	Chuck Table Dressing	Wafer Thinning Grinding
Grinding wheel	#600 (Grain size 18–21 μm)	(1) Rough grinding: #325 (Grain size 40–45 μm, resin bonder)
		(2) Fine grinding: #2000 (Grain size 4–5 μm, resin bonder)
Wheel rotation speed	1700 rpm	3000 rpm
Chuck table rotation speed	40 rpm	280 rpm
Final feeding speed	0.2 μm/s	0.3 μm/s
Coolant	Deionized water, 20 ± 0.5 °C	Deionized water, 20 ± 0.5 °C

## Data Availability

The data supporting reported results by the authors can be sent by e-mail.
